# A Patient with Rheumatoid Arthritis under Methotrexate and Etanercept Treatment Presenting with Fever and Pancytopenia: An Unexpected Guest

**DOI:** 10.31138/mjr.32.2.160

**Published:** 2021-05-28

**Authors:** Dimitrios Patoulias, Savvas Papachristou, Evgenia Gouridou, Dafni Stamou, Sofia Chissan, Spyridon Bakatselos

**Affiliations:** 1 First Department of Internal Medicine, General Hospital of Thessaloniki “Hippokration”, Thessaloniki, Greece; 2 Haematology Section, Second Propaedeutic Department of Internal Medicine, General Hospital of Thessaloniki “Hippokration”, Aristotle University of Thessaloniki, Thessaloniki, Greece

**Keywords:** Rheumatoid arthritis, leishmaniasis, anti-tumour necrosis factor-alpha, pancytopenia

## Abstract

Leishmania species induce chronic intracellular parasitism, while visceral leishmaniasis can become fatal, if left untreated. Patients with rheumatoid arthritis might feature a genetic predisposition to infection from Leishmania species, besides the status of immunosuppression. Several cases of visceral leishmaniasis in patients with underlying rheumatoid arthritis manifesting with cytopenias with or without organomegaly have been published so far; however, only three cases presenting with pancytopenia without splenomegaly have been described. Herein we describe a case of a 63-year-old woman presenting with fever and pancytopenia without organomegaly on a background of rheumatoid arthritis under methotrexate and etanercept treatment, finally diagnosed with visceral leishmaniasis.

## CASE REPORT

A 63-year-old woman, housewife, presented to the Emergency Department reporting protracted fever up to 38.5°C during the last two weeks (1–2 episodes per day) without accompanying symptoms except for weakness and anorexia, which did not respond after an empiric 4-day antibiotic treatment course with clarithromycin. Her past medical history included rheumatoid arthritis-with initial diagnosis 20 years ago- under prednisolone 5 mg twice daily, methotrexate 10 mg twice weekly, leflunomide 10 mg once daily, etanercept 50 mg once weekly, with coronary artery disease and hypertension. Patient was on prednisolone and methotrexate regimen during the last 5 years, while she was administered leflunomide and etanercept during the last year before admission, due to refractory disease. Physical examination did not reveal any pathological signs. Nevertheless, initial laboratory work-up revealed the presence of leukopenia (2700 white blood cells, with 1400 neutrophils per μL), normocytic-normochromic anaemia (35,8% haematocrit), thrombocytopenia (55000 per μL), as well as increased C-reactive protein (63 mg/L, reference value <6 mg/L), fibrinogen (410 mg/dL, reference range 180–350 mg/dL), erythrocyte sedimentation rate (84 mm/h), serum ferritin (845 ng/mL, reference range 10–291 ng/mL), and lactate dehydrogenase (368 U/L, reference value <248 U/L) levels.

**Figure 1.**
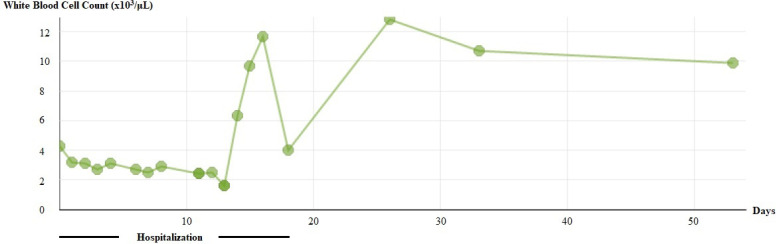
White blood cell count.

**Figure 2.**
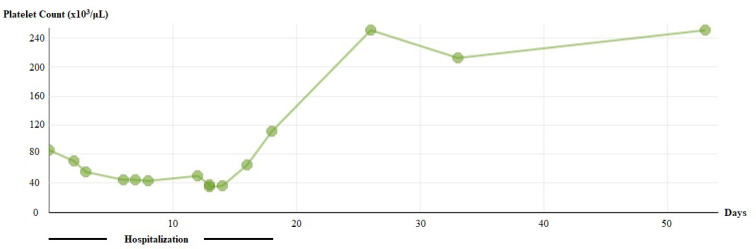
Platelet count.

**Figure 3.**
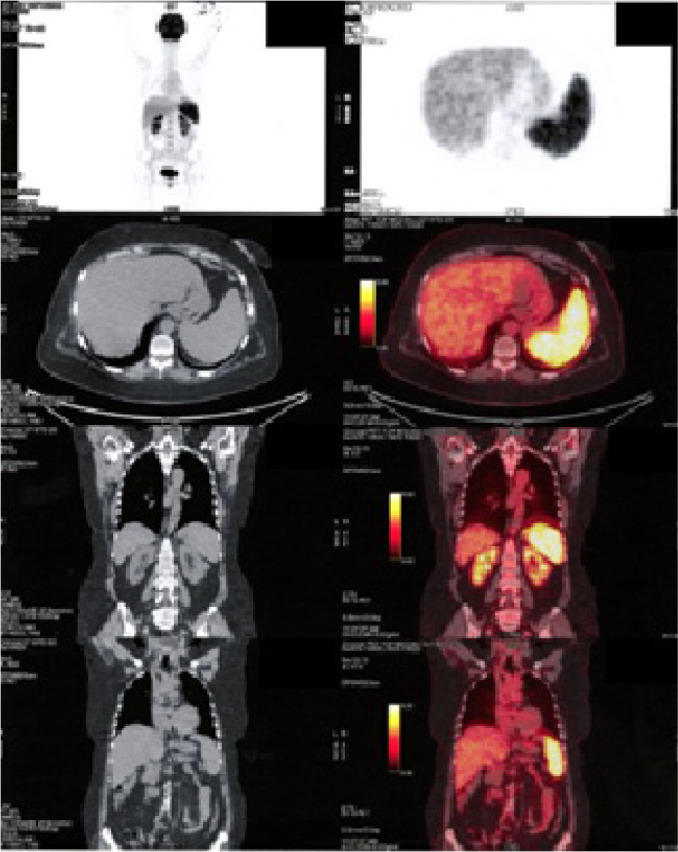
Positron emission tomography/computed tomography scan with fluorodeoxyglucose F18, showing increased metabolic activity of the spleen.

The patient was admitted for further investigation. She was placed on chemoprophylaxis with levofloxacin and doubling of prednisolone dose, while the rest of the rheumatoid arthritis medications were discontinued. An initial extensive laboratory work-up included blood and urine cultures, procalcitonin levels, testing for hepatitis B and C virus, human immunodeficiency virus, cytomegalovirus, Epstein-Barr virus, herpes simplex virus 1/2, Toxoplasma gondii, Parvo B19 virus, influenza A/B viruses, and respiratory syncytial virus, all of which turned out negative. The persistence of fever prompted a cervical, thoracic, and abdominal computed tomography scan, which did not reveal any remarkable findings. Due to a further reduction in the leukocyte count and increase in inflammatory markers, the antibiotic regimen was modified to piperacillin-tazobactam, teicoplanin and doxycycline. The lowest levels of neutrophils, platelets, and haematocrit that were recorded during her hospitalisation, were 700/μL, 35000/μL, and 29,6% respectively. The courses of her white blood cell and platelet counts are depicted in **Figure 1** and **Figure 2**, respectively. Myelogram, bone marrow biopsy, and serum protein electrophoresis were performed, revealing nonspecific findings, including non-specific IgGk hypergammaglobulinemia. Furthermore, blood smear and immunophenotype of peripheral blood, immunologic testing and levels of β2-microglobulin were also within normal range. Mild monocytosis was noticed. We finally requested for the conduction of a positron emission tomography/computed tomography scan with fluorodeoxyglucose F18. The latter displayed increased metabolic activity of the spleen, without splenomegaly (**Figure 3**). On that basis, further investigation for intra-cellular pathogens was decided, which revealed positive quantitative testing for Leishmania antibodies (enzyme-linked immunosorbent assay method). Notably, there were no epidemiologic data supportive of this diagnosis; however, clinical picture and laboratory results raised high index of clinical suspicion and prompted this investigation. The patient was finally diagnosed with visceral leishmaniasis and received treatment with liposomal amphotericin B at a dose of 3mg/kg/day on days 1–5, 14 and 21. Subsequent recession of fever within three days and gradual restoration of her laboratory parameters (**Figure 1** and **Figure 2**) were observed. She was discharged afebrile, in good general condition, while, two months later, she remains asymptomatic.

## DISCUSSION

After a comprehensive review of the available literature, we identified several cases of visceral leishmaniasis in patients with underlying rheumatoid arthritis manifesting with cytopenias with or without organomegaly (**Supplementary Table 1**); however, only three cases presented with pancytopenia without splenomegaly, while, only two occurred in the context of synchronous methotrexate and anti-tumour necrosis factor-alpha (anti-TNFa) treatment, as in our patient.^1–3^

Leishmania species cause chronic intracellular parasitism, while symptomatic disease in the immunocompromised patient usually occurs in the context of reactivation of a dormant infection.^4^ Major clinical features and laboratory findings of visceral leishmaniasis include fever, anorexia, weight loss, organomegaly, pancytopenia, and hypergammaglobulinemia.^4,5^ If left untreated, disease can lead to death within two years after its onset, due to severe anaemia or secondary bacterial infection.^5^ Interestingly, it can mimic autoimmune diseases, such as systemic lupus erythematosus^6^ and rheumatoid arthritis,^7^ inducing anti-CCP2 positivity.

According to recent data, there might be a genetic association between leishmaniasis and rheumatoid arthritis. Presence of the 469+14G/C polymorphism (rs3731865) of the SLC11A1 gene has been shown to increase the odds for developing rheumatoid arthritis by 60% [8], whereas s17221959, rs2279015, and rs17235409 polymorphisms are associated with visceral leishmaniasis.^9^ Further research towards this direction is required, in order to enable early recognition of prone patients.

Of note, TNFa blockade has been correlated with increased incidence of new cases of leishmaniasis, both mucocutaneous and visceral, as demonstrated in a recently published retrospective analysis, since TNFa is implicated into the initial pathophysiologic steps of infection.^10^ Thus, even in the presence of high index of clinical suspicion of leishmaniasis, anti-TNFa must be discontinued, while treatment should be re-evaluated, only when the infection fully resolves.

In conclusion, visceral leishmaniasis might be an atypical, opportunistic infection in patients with rheumatoid arthritis-even in non-endemic areas-, especially in those treated with anti-TNFa agents. Prompt discontinuation of immunosuppressive treatment along with early initiation of treatment of choice are the two major steps towards successful resolution of the infection and avoidance of complications.
